# Absence of DNA double strand breaks in human peripheral blood mononuclear cells after magnetic resonance imaging assessed by γH2AX flow cytometry: a prospective blinded trial

**DOI:** 10.1186/1532-429X-18-S1-O129

**Published:** 2016-01-27

**Authors:** Martin Fasshauer, Thomas Krüwel, Antonia Zapf, Vera Stahnke, Margret Rave-Fränk, Wieland Staab, Michael Steinmetz, Christina Unterberg-Buchwald, Andreas Schuster, Jan M Sohns, Christian O Ritter, Joachim Lotz

**Affiliations:** 1 Institute for Diagnostic and Interventional Radiology, University Medical Center Göttingen, Göttingen, Germany; 2 Department of Medical Statistics, University Medical Center Göttingen, Goettingen, Germany; 3 Department of Radiotherapy and Radiooncology, University Medical Center Göttingen, Goettingen, Germany; 4 Departmet of Pediatric Cardiology and Intensive Care, University Medical Center Göttingen, Goettingen, Germany; 5 Department of Cardiology and Pneumology, University Medical Center Göttingen, Goettingen, Germany

## Background

Magnetic Resonance Imaging (MRI) is regarded as a non harming and non invasive imaging modality with superb tissue contrast and almost no side effects. Compared to other cross-sectional imaging modalities MRI does not use ionizing radiation. Recently however, strong magnetic fields as applied in clinical MRI scanners have been suspected to induce DNA double strand breaks in human lymphocytes.

## Methods

In this study we investigated the impact of 3 T cardiac MRI examinations on the induction of DNA double strand breaks in peripheral mononuclear cells by ɣH2AX staining and flow cytometry analysis. The study cohort consisted of 73 healthy non-smoking volunteers with 36 volunteers undergoing CMRI and 37 controls without intervention. Differences between the two cohorts were analysed by a mixed linear model with repeated measures.

## Results

Both cohorts showed a significant increase of the ɣH2AX signal from baseline to post procedure of 6·7% and 7·8%, respectively. The difference between the two groups was not significant.

## Conclusions

We therefore conclude that clinical 3 T MRI as used in cardiac imaging does not have an impact on DNA integrity.

**Table 1:** Relative change of the mean fluorescence intensity (MFI) from baseline to post procedure. Both groups showed a significant increase in MFI. The difference between both groups was not significant. SD: standard deviation.

group	N	relative increase MFI	95%-confidence interval	SD	p-value
Control	37	7.80%	5.6 - 10.0	6.61	< 0.001
MRI	36	6.73%	4.30 - 9.16	7.18	< 0.001

